# Functional and Behavioral Responses of the Natural Enemy *Anthocoris nemoralis* to *Cacopsylla pyri*, at Different Temperatures

**DOI:** 10.1007/s10905-023-09836-5

**Published:** 2023-07-07

**Authors:** Laura A. Reeves, Michael P. D. Garratt, Michelle T. Fountain, Deepa Senapathi

**Affiliations:** 1 Centre for Agri-Environmental Research, School of Agriculture, Policy and Development, University of Reading, Reading, Berkshire RG6 6AR UK; 2 NIAB, New Road, East Malling, Kent, ME19 6BJ UK

**Keywords:** *Anthocoris nemoralis*, anthocorids, pear sucker, temperature, functional response, behavioral assays.

## Abstract

*Anthocoris nemoralis* is the dominant predator of pear sucker (*Cacopsylla pyri*) in the UK. *Anthocoris nemoralis* migrates into orchards in spring or is introduced as a biocontrol agent, reaching peak population levels in July-August, contributing to effective control of summer pear sucker populations. However, due to temperature dependent development and metabolism there are concerns that *C. pyri* populations or feeding rates may increase due to changing climatic conditions. Thus, how *A. nemoralis* responds to temperature, impacts its ability as a biocontrol agent. Functional response assays, monitoring attack rate and handling time of *A. nemoralis* and behavioral assays, using Ethovision tracking software occurred, to assess the impact of temperature on predation. Experiments were conducted at current and future July-August mean temperatures, predicted using RCP4.5 and RCP8.5 (medium and high, representative concentration pathway) emissions scenarios, using 2018 UK Climate Projections (UKCP18). All treatments demonstrated a Type II functional response, with female anthocorids demonstrating shorter handling times and higher attack rates than males. Males showed longer prey handling times at 18 °C compared to 23 °C and more time was spent active at lower temperatures for both sexes. Females did not show significant differences in attack rate or handling time in response to temperature. Overall prey consumption was also not significantly affected by temperature for either sex. This study suggests that anthocorids are likely to remain effective natural enemies under future predicted temperatures, due to non-significant differences in prey consumption.

**Supplementary Information:**

The online version contains supplementary material available at 10.1007/s10905-023-09836-5.

## Introduction

The anthocorid, *Anthocoris nemoralis* (Fabricius), is the main natural enemy of pear sucker (*Cacopsylla pyri* L.) in the UK and Europe (Solomon et al. 2000; Nagy et al. 2008; Sigsgaard 2010). The estimated cost of pear sucker to the UK pear industry is £5 million per annum in damage and control costs (AHDB 2012). These phloem feeding insects damage pear trees in three main ways: nymphs produce honeydew, a sugary secretion that encourages the growth of black sooty mold (Daniel et al. 2005; Salvianti et al. 2008; Montanari et al. 2015), adult *C. pyri* are a vector of pear decline disease (*Candidatus* Phytoplasma pyri); which reduces shoot and fruit growth in pear and can lead to tree death (Carraro et al. 2001; Kucerová et al. 2007; Süle et al. 2007) and high numbers of *C. pyri* can cause ‘psylla shock’; toxic saliva is injected into pear leaves, resulting in defoliation and fruit drop (Erler 2004; Saour et al. 2010; Oz and Erler 2021). With a high resistance to commonly available pesticides (Erler 2004; Sek Kocourek and Stará 2006) many growers currently practice integrated pest management (IPM) of pear sucker, focusing on maximizing natural enemy populations, to control pear sucker (Shaw et al. 2021). Natural migrations of anthocorids can reduce pear sucker populations during the summer (Nagy et al. 2008). Adult *A. nemoralis* often overwinter in hedgerows or on unmanaged vegetation, migrating into orchards in April-May to lay eggs, when pear sucker populations are increasing (Shaltiel and Coll 2004; Nagy et al. 2008). *Anthocoris nemoralis* populations usually peak during July-August, helping to control *C. pyri* numbers (Fields and Beirne 1973; Scutareanu et al. 1999). However, anthocorids can also be released artificially into orchards as a biocontrol agent, to reduce pear sucker populations more rapidly (Beninato and Morella 2000; Gajski and Pekár 2021). Nymphs and adult *A. nemoralis* predate upon pear sucker eggs and nymphs (Sigsgaard 2010) and have a pierce-sucking stylet to feed (Bulgarini et al. 2021). A single anthocorid is estimated to consume almost 5000 eggs during its lifetime (Yanik and Ugur 2004), with no significant preference shown between eggs and nymphs based on biomass (Sigsgaard 2010).

There is increasing concern that rising temperatures may impact pest populations (Barford 2013; Sable and Rana 2016; Zidon et al. 2016). Insects are poikilothermic, this means they have a body temperature that fluctuates with their environment (May 1979; Sable and Rana 2016; Wojda 2017). Therefore, rising temperatures could impact pest development (Ratte 1984; Campolo et al. 2014), fecundity (Kindlmann et al. 2001; Boggs 2016), number of generations per year (Tobin et al. 2008), overwintering times (Ladányi and Horváth 2010) and behavior (Mellanby 1939). Pear sucker have temperature dependent development (Kapatos and Stratopoulou 1999; Schaub et al. 2005); faster development rates at warmer temperatures could lead to shorter generation times, potentially increasing pest populations. There is concern that warmer temperatures could alter the feeding behavior, activity and fecundity of phloem feeders (McMullen and Jong 1972; Liu et al. 2021). One explanation for increased feeding rate under high temperatures is due to altered metabolism (Yuan et al. 2009), as metabolic rate increases exponentially with temperature up to a certain threshold, increasing demand for energy and nutrients (Schmitz and Barton 2014; Frances and McCauley 2018). Furthermore, the scale of metabolic increase is largely dependent on body size, with smaller species having higher increases in metabolism than larger species (Frances and McCauley 2018). Thus, as prey species are often smaller than their predators, their metabolism may increase at a faster rate with respect to warming, leading to an enhanced feeding rate. For example, *C. pyri* adults are less than 3 mm and nymphs in their 5th instar are 1.9 mm in length (Chireceanu 1998), compared to *A. nemoralis* adults which have a body length of 3.5–4 mm (BPDB 2022). Therefore, feeding rates of pear sucker prey may increase more than their anthocorid predators due to body size.

It is important, therefore, to establish whether the feeding rate of *A. nemoralis* increases with temperature, to understand if it will be an efficient natural enemy of *C. pyri* under future predicted temperature conditions. One of the most effective ways of monitoring predator-prey interactions is by fitting functional responses; a functional response can be defined as the change in the consumption rate for a predator depending on prey density, therefore whether it is density dependent (Holling 1965; Real 1977). The functional response of a predator is determined by different parameters including: attack rate ($a$); rate of discovery of a prey item and handling time ($h$); the time period when the predator consumes its prey (including killing, capturing, eating and sometimes digesting) (Real 1977; Juliano 2020). There are three main “Types” of functional responses, which all have different shapes and can be defined as follows: a Type I response shows a linear increase in consumption rate depending on prey density (up to a certain threshold), as the time needed to consume or process prey is negligible (Real 1977; Jeschke et al. 2004; DeLong 2021). A Type II functional response differs from a Type I functional response as it includes handling time ($h$); a time period when the predator consumes its prey (including killing, capturing, eating and digesting), therefore to begin with consumption rate of prey increases as prey density increases, but eventually levels off and remains constant at high densities (Real 1977; Juliano 2020). Type III functional response resembles Type II at high prey densities; however at low densities the consumption rate of a predator increases slowly, due to learning time or prey switching, giving the response curve a sigmoidal shape (Real 1977; DeLong 2021).

In biological control, Type I functional responses are scarce as they are almost exclusive to filter feeders, as handling time is rarely negligible in other species (Real 1977; Jeschke et al. 2004). Having a Type II functional response is more optimal than a Type III for a biological control agent, as natural enemies are still able to detect and attack pests at low densities (Lopes et al. 2009). However, Type III responses allow a negative density-dependent response of the prey survival with prey population density, compared to type II which may help stabilize prey populations, making them less likely to fluctuate (Cuthbert et al. 2021). Functional responses are also influenced by multiple biotic and abiotic factors including; life-stage of predator or prey (Farhadi et al. 2010), sex of predator (Emami et al. 2014a), species of prey (Milonas et al. 2011), temperature (Englund et al. 2011) and arena size (Uiterwaal and DeLong 2018). Although functional responses can be largely influenced by temperature (Englund et al. 2011), there are no studies to date on the natural enemy *A. nemoralis*, using the prey species *C. pyri*, at multiple temperature regimes. Although, other functional response experiments have occurred on other anthocorid species (Kheradmand et al. 2017; Hassanzadeh-Avval et al. 2019)d nemoralis (Emami et al. 2014b) at a single temperature (27 °C), allowing comparison.

Changes in behavioral responses are important when monitoring trophic interactions between predator and prey (Chen et al. 2015; Duffy et al. 2015; Boege et al. 2019). For example, changes in walking velocity or distance travelled by a natural enemy could alter the probably of encountering a prey item or host (Milton 2004). Whilst changes in cleaning/grooming behavior may increase risk of disease; as grooming is an important sanitary behavior, involved in the removal of pathogens (Zhukovskaya et al. 2013).

This study aims to monitor the behavior and functional response of the natural enemy, *A. nemoralis*, to determine whether it would be an efficient biological control agent under future UK predicted temperatures. Behavioral and functional responses were monitored at three temperature regimes (18 °C, 21 and 23 °C) selected based on current mean July-August temperatures and mean temperatures predicted for July-August by RCP 4.5 and RCP 8.5 emissions scenarios for 2080. Our study tested four hypotheses: (1) Anthocorids demonstrate a Type II functional response, (2) Handling time is shortened and attack rates are increased at higher temperatures, (3) Behaviors including movement, feeding and cleaning of anthocorids increase under elevated temperatures and (4) The sex of anthocorid impacts the functional response, with shorter handling times and higher attack rates for females, due to a larger body size.

## Materials and Methods

### Pear Sucker and Anthocorid Husbandry

Pear sucker nymphs hardshell nymphs (L4-L5, the fourth or fifth nymph stage in a pear sucker’s life history) were collected from cv. Conference pear trees (*Pyrus communis*) at NIAB East Malling (51.2885° N, 0.4383° E). Nymphs were removed from trees daily, using a soft, fine tipped paintbrush, to minimize damage to insects. These nymphs were used for functional response experiments and behavioral assays. Adult *C. pyri* were collected using beat tray sampling. A pear tree branch was tapped with a foam-covered stick, with a white tray (260 mm by 460 mm) held underneath. Adult *C. pyri* were kept in ventilated Tupperware pots (diameter 94.7 mm, height 115 mm) containing 3 pear shoots (110 mm in length) in damp tissue. Individuals were kept in a controlled temperature (CT) cabinet at 21 °C. Semi-mature pear sucker eggs (yellow-white in color) were collected from the Tupperware pots daily, these were used for the egg treatment within behavioral assays. *C. pyri* adults and nymphs were identified to species level using the Psyllid key from RLP Agroscience (Agroscience 2022).

A batch of 500 *A. nemoralis* adults were ordered from the biocontrol company Koppert each week of the study. This product was called Anthobug and ordered from Koppert UK Ltd, Suffolk, CB9 8PJ. These were approximately 4–10 days after their final molt, when used in behavioral and functional response experiments. Anthocorids were kept in a ventilated plastic container, with the carrier material they arrived in and fed *C. pyri* eggs daily. Individuals were allowed to mate, with males and females kept in the same container in a CT cabinet at 21 °C. Five batches of anthocorids were used for behavioral response assays and seven batches for functional response experiments. Following a Kruskal Wallis test no significant difference was found between batches depending on velocity, distance travelled, number of *C. pyri* eggs or nymphs eaten or time spent exhibiting a behavior, apart from cleaning, where there was a significant difference in the time spent cleaning between batch 3 and 5 (Table S1). Therefore, batch number was not included within models. Male and female anthocorids were identified using a light microscope based on differences in genitalia. If there was some uncertainty in the sex of the anthocorid, individuals were dissected after the experiment to find parameres, if male (Hassanzadeh-Avval et al. 2020), or copulatory tubes if female (Ke and Bu 2007).

### Functional Response Experiments

For functional response experiments, adult *A. nemoralis* were starved for approximately 24 h at either 18 °C, 21 or 23 °C in controlled temperature (CT) cabinets. Then, a male or female individual was added to a triple-vented Petri dish (55 mm in diameter). The floor of the dish was covered with 1% set agar to provide moisture and support for leaf disks as used in the functional response experiments of Hassanzadeh-Avval et al. (2019). The Petri dish contained a leaf disk of *P. communis* ‘Conference’ (20 mm in diameter) and *C. pyri* nymphs (4th − 5th instar), at one of five densities (5, 10, 15, 30 and 50 nymphs).

After the anthocorid was added, the Petri dish was sealed with plastic paraffin film to prevent *C. pyri* nymphs escaping (similar to Emami et al. (2014a)) and returned to the same temperature treatment for 24 h. Nymphs were not replaced during the experiment. After 24 h the anthocorid and numbers of *C. pyri* nymphs were recorded as alive or dead. There were 10 replicates for *A. nemoralis* male and female tests at each temperature treatment, for the 5 prey densities, giving a total of 300 observations. Five control treatment replicates of *C. pyri* nymphs were set up for each temperature, to quantify natural mortality.

### Behavioral Assays

Similar to the functional response experiments, adult *A. nemoralis* were starved for 24 h, in one of the three temperature treatments (18 °C, 21 and 23 °C) in CT cabinets. After this the anthocorid was moved to a CT room with insect behavior tracking software; Ethovision (Noldus et al. 2001, 2002). The anthocorid was then added to a triple-vented Petri dish (55 mm in diameter). The Petri dish contained a 5 mm piece of leaf with either, 2 *C. pyri* nymphs (4-5th instar), 15 semi-mature *C. pyri* eggs, or no prey (as a control). The numbers of eggs and nymphs were chosen as they were approximately equivalent to each other in size. The leaf containing the food was placed in the center of the Petri dish (marked with a cross), the anthocorid was then placed in the 20 mm center circle (not on the leaf) and given 10 min to acclimatize. After this period the Ethovision camera was set to record for 20 min, then the anthocorid was removed and the number of nymphs/eggs consumed were counted.

Movement and behaviors of anthocorids were recorded using Ethovision XT tracking software (Noldus et al. 2001, 2002); the velocity, distance travelled, time spent in the center (20 mm diameter center circle) and edge (up to 10 mm from the edge) zones and time spent displaying certain behaviors were recorded. These measurements were tracked from the center-point of the anthocorid’s body. The 6 recorded behaviors were, feeding (when the anthocorid was stationary and had its stylet in an egg or nymph), moving (when the anthocorid was walking or flying), moving leaf (when the anthocorid was moving the leaf around the arena), antennating (when the anthocorid was stationary and repeatedly touching a surface with its antennae), cleaning (when an anthocorid was grooming its legs or antennae) and stationary (when an anthocorid was completely still and not feeding). All behaviors were independent of each other, for example an anthocorid that was stationary could not also be cleaning. There were ten replicates each of the three food treatments, three temperature treatments and if the anthocorid was male or female, giving a total of 180 observations.

### Temperature Regimes and Controlled Temperature Cabinets

The three temperature treatments (18, 21 or 23 °C) were determined based on the current mean July-August temperature (1990–2020) and mean July-August temperatures predicted in 2080, based on the RCP4.5 (medium emissions) and RCP8.5 (high emissions) scenarios. A 2080 time frame was chosen as this year is commonly used in studies predicting future trophic interactions (Duffy 2014; South et al. 2018; Aartsma et al. 2019), thus the results of this paper can be compared to others. July-August temperatures were chosen as this is when anthocorids are most abundant in pear orchards (Fields and Beirne 1973; Scutareanu et al. 1999). The current temperature was calculated using mean July-August temperatures (1990–2020) from East Malling weather station (51.288° N, 0.448° E) in Kent. To calculate future temperatures for 2080, data was extracted using the UK Climate Projections User Interface, based on UKCP18 projections (UKCP 2021). The predicted increase in mean air temperature at 1.5 m for 2080 was calculated for July to August (baseline scenario 1981–2000) for a 25 km x 25 km region in Kent, surrounding East Malling (562500.00, 162500.00), these temperatures were calculated for each of the RCP4.5 and RCP8.5 scenarios and added to the average 1981–2000 July-August temperature (17.41 °C). The predicted temperatures were rounded up to the nearest degree (Table S2), as many functional response experiments use temperatures to the nearest degree (Ding-Xu et al. 2007; Hassanzadeh-Avval et al. 2019; Hassanpour et al. 2020), allowing comparison.

The three controlled temperature (CT) cabinets (set at 18, 21 and 23 °C) had two containers half-filled with water to keep humidity constant (Table S2). Temperature and humidity were monitored using EasyLog USB dataloggers (Table S2). The daylight cycle within the cabinets was 16 h light, 8 h dark, based on average summer day length in the UK.

### Data Analyses

#### Functional Response Experiments

The type of functional response for each of the treatments (sex and temperature) was selected and fitted using the R package FRAIR (Pritchard et al. 2017). This method involved three different steps: model selection, model fitting and model comparison. Firstly, polynomial logistic functions were fitted to the data to identify the ‘Type’ of functional response (Type I, Type II or Type III), as outlined by Juliano et al. (2001). Within a logistic regression a Type II functional response can be identified by a negative first-order term (where prey consumption is negatively proportional to prey density), whereas a Type III functional response has a positive first-order term. The frair-test function within the ‘FRAIR’ package in R was used for model selection; classifying the type of functional response, based on the sign and significance of first-order and second-order terms within logistic regressions (Pritchard et al. 2017). For model fitting the frair-fit function was used. This function undertakes optimization by maximum likelihood estimation (MLE), giving information on the model fit, maximum likelihood estimators and a regression output.

Due to the fact resources were being depleted throughout the experiment (nymphs were not replaced when eaten), the Rogers Random predator equation was used (Rogers 1972), as this equation is applicable to non-replacement experiments and is solved via the ‘lambertW’ function (Bolker 2008; Pritchard et al. 2017; DeLong 2021). For all Type II functional responses within this experiment the following equation was used:

1 $${N}_{a}= {N}_{0}[1-\text{exp}\left(a\left({T}_{h}{N}_{a}-T\right)\right)]$$

${N}_{a}$ is the number of prey (pear sucker nymphs) consumed by the predator (anthocorid), ${N}_{0}$ is the number of prey initially offered to the predator, $a$ is the attack rate, ${T}_{h}$ is the handling time and $T$is the time in hours that the prey are exposed to the predator (24 h).

Finally, the functions frair-compare and frair-boot within the FRAIR package were used to compare differences between temperature treatments and sex of anthocorid. The frair-compare function used a difference test, with the null hypothesis that fitted parameters ${D}_{a}$ (difference in attack rate) and ${D}_{h}$ (difference in handling time) do not differ, depending on treatment (temperature and sex). The frair-boot function uses nonparametric bootstrapping, generating 95% confidence intervals (CI) for attack rate ($a$) and handling time ($h$), to see if CIs overlap between treatments. These bootstrap outputs for 95% CIs were plotted using the function drawpoly. The difference between number of prey eaten depending on density at the 3 different temperatures was tested using a Kruskal-Wallis test, as data were non-normally distributed.

### Behavioral Assays

For behavioral response assays, stacked bar charts were created using the ‘ggplot2’ package in R. Stacked barcharts displayed the percentage of time *A. nemoralis* spent demonstrating certain behaviours (feeding, moving, moving leaf, cleaning, stationary and antennating) over the 20-minute time period, for three different temperatures and three food treatments. Heatmaps were created to show the proportion of time *A. nemoralis* spent in the center zone (containing food/leaf), middle and edge of the arena. Heatmaps were created using Ethovision XT tracking software (Noldus et al. 2001). For statistical analysis a Kruskal-Wallis H test, followed by pairwise comparisons using a Wilcoxon rank sum test, as data were non-normally distributed. These tests were used to compare differences in behavior, time spent in zones, velocity and distance travelled, depending on treatment.

## Results

### Functional Response Experiments

For the control experiments, without an anthocorid present, there was an average of 0.43 dead nymphs per sample. This ranged from 0 deaths in the 5-nymph density, to 1.27 ± 1.62 deaths in the 50 nymph density. The number of dead nymphs in the control experiment was significantly lower compared to the corresponding treatments containing anthocorids (χ^2^ = 174.01, df = 5, p < 0.001), based on a Kruskal-Wallis test with pairwise comparisons using the Wilcoxon rank sum test. Therefore, it was likely that the anthocorids were causing nymph deaths rather than other factors. The number of nymphs eaten significantly differed depending on density; Kruskal Wallis: χ^2^ = 126.97, df = 4, p < 0.001. With an average of 3.82 ± 1.14 nymphs eaten over 24 h at the lowest density and an average of 10.02 ± 3.57 nymphs eaten at the highest density. However, the Wilcoxon rank sum test indicated that the number of nymphs eaten at the density 30 did not significantly differ from the density of 50 (*p* = 0.285), suggesting that the saturation point for prey consumption had been reached. This non-significant difference between 30 and 50 nymphs occurred for both male and female anthocorids. There was a significant difference in number of nymphs eaten depending on sex of anthocorid; Kruskal Wallis: χ^2^ = 66.51, df = 1, p < 0.001, with an average of 11.97 ± 3.41 nymphs eaten by females at the highest density and an average of 8.07 ± 2.45 eaten by males. However, the number of nymphs eaten depending on temperature did not significantly differ for females (Kruskal Wallis: χ^2^ = 2.44, df = 2, *p* = 0.296) or males (Kruskal Wallis: χ^2^ = 1.70, df = 2, *p* = 0.427), with an average of 9.85 ± 4.13 nymphs killed at 18 °C, 9.90 ± 2.99 nymphs at 21 °C and 10.30 ± 3.66 nymphs at 23 °C, for the highest prey densities.

**Fig. 1:**
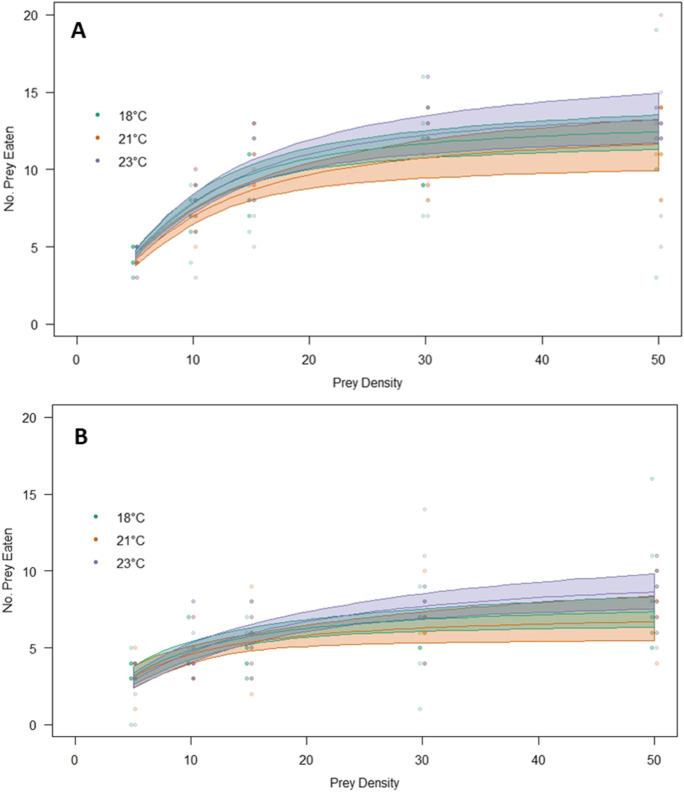
The number of prey eaten (*Cacopsylla pyri* nymphs) by the predator *Anthocoris nemoralis*, depending on prey density. For both **A** female and **B** male *Anthocoris nemoralis*, at three different temperature regimes (18 °C, 21 and 23 °C). Type II functional response curves based on bootstrapped model fits for 95% confidence intervals

**Table 1:** Evidence for Type II or Type III functional responses using the frair-test function, for different temperature treatments (18 °C, 21 and 23 °C) and male or female *Anthocoris nemoralis*

Temperature (°C)	Sex	Estimate (density)	SE	Z value	P value	Evidence for Type II response
18	M	-0.038	0.0045	-8.28	**< 0.0001**	Yes
21	M	-0.039	0.0045	-8.82	**< 0.0001**	Yes
23	M	-0.037	0.0044	-8.38	**< 0.0001**	Yes
18	F	-0.048	0.0042	-11.39	**< 0.0001**	Yes
21	F	-0.059	0.0044	-13.43	**< 0.0001**	Yes
23	F	-0.056	0.0043	-13.01	**< 0.0001**	Yes

This method uses forward selection based on the sign and significance of first (density) and second-order terms within logistic regressions. A significant negative estimate of density provides evidence for Type II response. Entries in bold show significant *p* values

During model selection a Type II functional response was chosen for both male and female anthocorids, for all three temperature regimes, due to the fact the first order term (density) from logistic regressions was negative and significant for all treatments (Table 1; Fig. 1).

Attack rates ($a$) generated from the maximum likelihood optimisation output ranged from 0.049 (23 M) to 0.156 (21 F). Whilst handling time ($h$) ranged from 3.27 (18 M) to 1.65 (23 F) (Table 2). The maximum number of nymphs that were attacked per day $\left(T/{T}_{h}\right)$was 14.54 (23 F) and the minimum number was 7.34 (18 M).

**Table 2:** Maximum likelihood estimates (MLE) of attack rate ($a$) and handling time ($h$) and their standard errors (SE), for male and female *A. nemoralis* at three different temperature treatments (18 °C, 21 and 23 °C)

Temp (°C)	Sex	Coeff	Estimate	SE	Z value	P value	Response	Log L
18	M	$a$	0.069	0.020	3.51	**< 0.0001**	Rogers Type II	200.28
		$h$	3.27	0.361	9.06	**< 0.0001**		
21	M	$a$	0.069	0.018	3.77	**< 0.0001**	Rogers Type II	190.74
		$h$	2.95	0.325	9.08	**< 0.0001**		
23	M	$a$	0.049	0.010	5.12	**< 0.0001**	Rogers Type II	205.57
		$h$	2.33	0.266	8.78	**< 0.0001**		
18	F	$a$	0.108	0.019	5.82	**< 0.0001**	Rogers Type II	199.85
		$h$	1.85	0.146	12.64	**< 0.0001**		
21	F	$a$	0.156	0.025	6.26	**< 0.0001**	Rogers Type II	191.61
		$h$	1.78	0.116	15.41	**< 0.0001**		
23	F	$a$	0.135	0.021	6.50	**< 0.0001**	Rogers Type II	218.78
		$h$	1.65	0.114	14.43	**< 0.0001**		

Logistic regressions use a Rogers Type II response, as prey is not replaced during the experiment. Entries in bold show significant *p* values

**Table 3:** Comparisons of male and female *Anthocoris nemoralis* at three different temperature treatments (18 °C, 21 and 23 °C)

Comparison	Coefficients	Estimate	SE	Z value	P value	Response
18 F ~ 18 M	${D}_{a}$	0.039	0.027	1.43	0.152	Rogers Type II
	${D}_{h}$	-1.43	0.390	-3.66	**0.0003**	
21 F ~ 21 M	${D}_{a}$	0.087	0.031	2.83	**0.0047**	Rogers Type II
	${D}_{h}$	-1.17	0.345	-3.40	**0.0007**	
23 F ~ 23 M	${D}_{a}$	0.086	0.023	3.76	**0.0002**	Rogers Type II
	${D}_{h}$	-0.68	0.289	-2.37	**0.0179**	

Using the difference method, with difference in attack rate $\left({D}_{a}\right)$ and difference in handling time $\left({D}_{h}\right)$, p values in bold show a significant difference

**Table 4:** Comparisons of three different temperature treatments (18 °C, 21 and 23 °C), for male and female *Anthocoris nemoralis*

Temperature	Coefficients	Estimate	SE	Z value	P value	Response
18 M ~ 21 M	${D}_{a}$	0.0003	0.027	0.013	0.990	Rogers Type II
	${D}_{h}$	0.317	0.487	0.652	0.515	
18 M ~ 23 M	${D}_{a}$	0.020	0.022	0.907	0.365	Rogers Type II
	${D}_{h}$	0.946	0.449	2.11	**0.035**	
21 M ~ 23 M	${D}_{a}$	0.020	0.021	0.957	0.339	Rogers Type II
	${D}_{h}$	0.631	0.420	1.50	0.133	
18 F ~ 21 F	${D}_{a}$	-0.048	0.031	-1.54	0.124	Rogers Type II
	${D}_{h}$	0.066	0.186	0.353	0.724	
18 F ~ 23 F	${D}_{a}$	-0.028	0.028	-0.987	0.324	Rogers Type II
	${D}_{h}$	0.203	0.185	1.10	0.273	
21 F ~ 23 F	${D}_{a}$	0.020	0.032	0.619	0.536	Rogers Type II
	${D}_{h}$	0.138	0.162	0.847	0.397	

Using the difference method, with difference in attack rate $\left({D}_{a}\right)$ and difference in handling time $\left({D}_{h}\right)$, p values in bold show a significant difference

For model comparison there was a significant difference between male and female anthocorids for all temperature treatments (Table 3). The difference in attack rate $\left({D}_{a}\right)$ and handling time $\left({D}_{h}\right)$ was significant for the 23 F ~ 23 M and 21 F ~ 21 M comparisons, however for the 18 F ~ 18 M comparison only ${D}_{h}$was significant. For comparisons between temperatures, 23 M had a significantly lower handling time than 18 M (Table 4). However, there were no significant differences for any of the other temperatures. For the bootstrapping method, CIs for $a$ and $h$ overlapped for all different temperature treatments (Table S3). However, the CIs for $a$ did not overlap between male and female anthocorids, at higher temperature treatments.

### Behavioral Assays

#### Velocity and Distance Travelled

There was a significant difference in the average velocity of *A. nemoralis* depending on food treatment (Kruskal Wallis: χ^2^ = 61.10, df = 2, *p* > 0.001), the average velocity was significantly lower for the nymph treatment (0.069 cm/s ± 0.14) compared to eggs (0.16 cm/s ± 0.14) and no food (0.22 cm/s ± 0.26). Highlighting that the velocity of the anthocorid is significantly higher when no food is present. This was also similar for average distance travelled by *A. nemoralis* depending on food treatment (Kruskal Wallis: χ^2^ = 61.53, df = 2, *p* > 0.001), the average distance travelled was significantly lower for the nymph treatment (80.79 cm ± 164.9) compared to eggs (184.1 cm ± 163.1) and no food (308.0 cm ± 265.9). There was no significant difference in velocity (Kruskal Wallis: χ^2^ = 0.066, df = 1, *p* = 0.797) or distance travelled (Kruskal Wallis: χ^2^ = 0.075, df = 1, *p* = 0.784) depending on the sex of anthocorid, nor velocity (Kruskal Wallis: χ^2^ = 3.44, df = 2, *p* = 0.179) or distance travelled (Kruskal Wallis: χ^2^ = 3.41, df = 2, *p* = 0.181) depending on temperature.

#### Time Spent in Different Zones

**Fig. 2:**
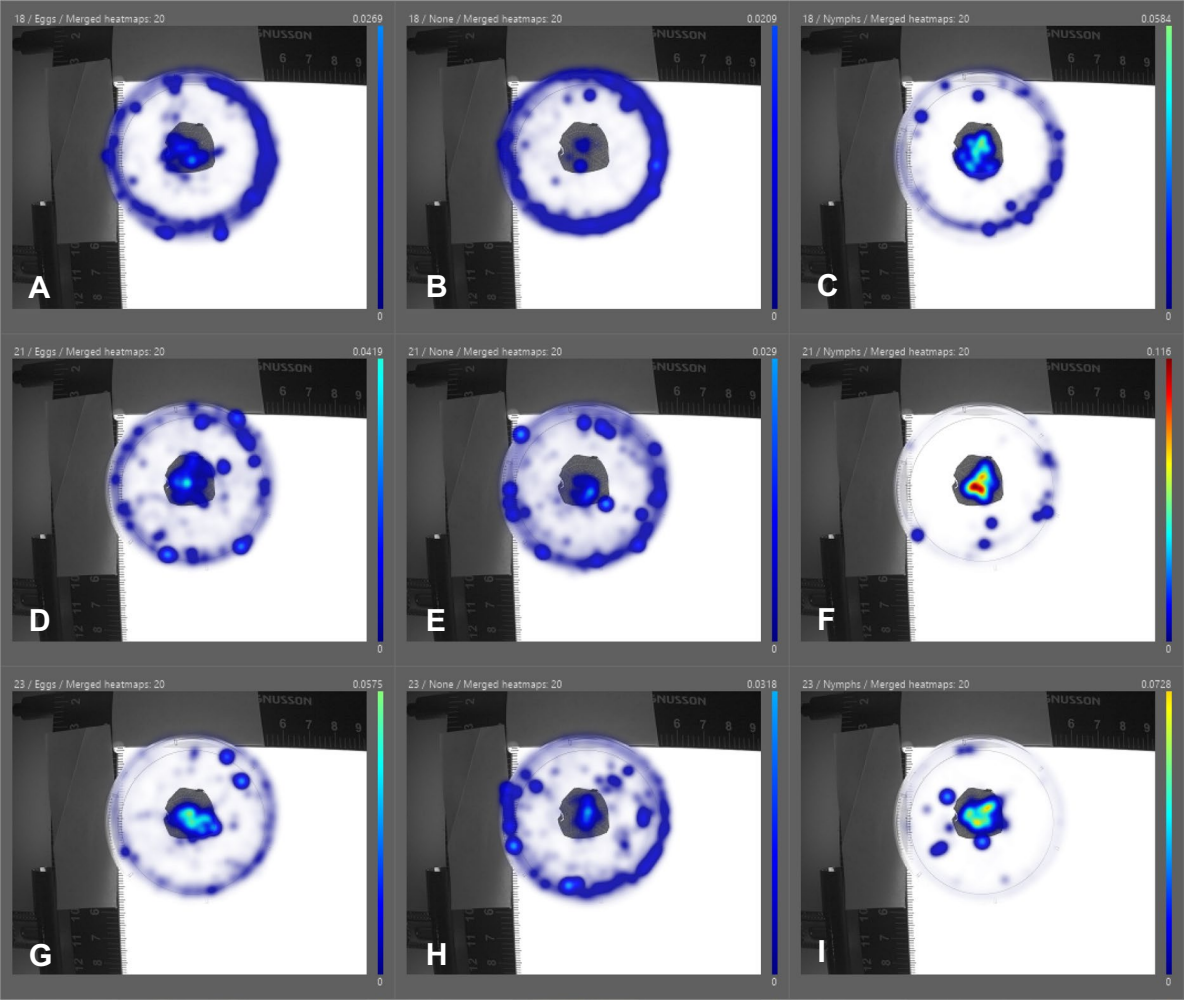
The proportion of time spent by the anthocorid predator *Anthocoris nemoralis* in different areas within the arena (55 mm in diameter), depending on temperature treatment (**A- C.**18 °C, **D-F.** 21 °C and **G-I.** 23 °C) and food type (**A, D, G.** eggs, **B, E, H.** no-food and **C,F,I.** nymphs) for *Cacopsylla pyri* prey. The center zone is marked out by the grey circle (20 mm in diameter)

There was a significant difference in the time *A. nemoralis* spent in the center zone depending on food source (Kruskal Wallis: χ^2^ = 58.04, df = 2, *p* > 0.001), the average time spent was significantly higher for the nymph treatment (945.2 s ± 439.9) compared to eggs (488.9 s ± 476.8) and no food (159.0 s ± 303.4) (Figs. 2 and 4).

**Fig. 3:**
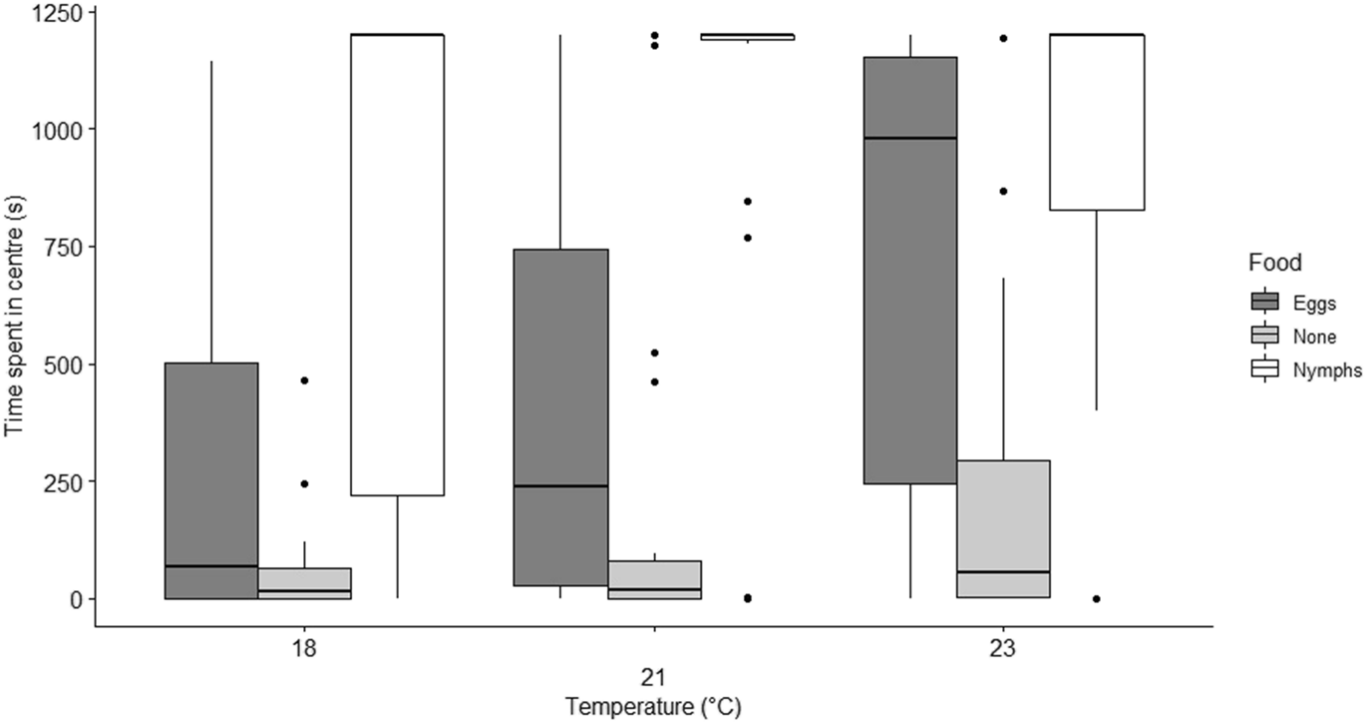
The amount of time (s) spent in the center of the arena (20 mm in diameter) containing the food source or leaf, for the 20-minute (1200s) time period, depending on temperature treatment (18 °C, 21 and 23 °C) and food type (eggs, no-food and nymphs) for the anthocorid predator *Anthocoris nemoralis* for *Cacopsylla pyri* prey

There was also a significant difference in time spent in the edge zone depending on food source (Kruskal Wallis: χ^2^ = 45.32, df = 2, *p* > 0.001), the average time spent was significantly higher for the no-food treatment (452.1 s ± 327.1) compared to eggs (297.7 s ± 307.74) and nymphs (132.0 s ± 273.9). There was no significant difference between the amount of time spent in the center (Kruskal Wallis: χ^2^ = 0.735, df = 2, *p* = 0.391) or edge (Kruskal Wallis: χ^2^ = 0.003, df = 2, *p* = 0.956) zones depending on sex of the anthocorid. For the egg treatment, there was a significant difference in time spent in the center zone depending on temperature (Kruskal Wallis: χ^2^ = 10.91, df = 2, *p* = 0.004), with significantly more time spent in the center zone (Figs. 2 and 3) in the 23 °C (753.5 s ± 478.8) treatment compared to 21 °C (422.4 s ± 442.5) and 18 °C (290.8 s ± 401.6). However, there was no significant difference for time spent in center zone depending on temperature for the nymphs (Kruskal Wallis: χ^2^ = 1.09, df = 2, *p* = 0.580) or no-food treatment (Kruskal Wallis: χ^2^ = 1.58, df = 2, *p* = 0.455).

### Behaviors Demonstrated and Prey Eaten

**Fig. 4:**
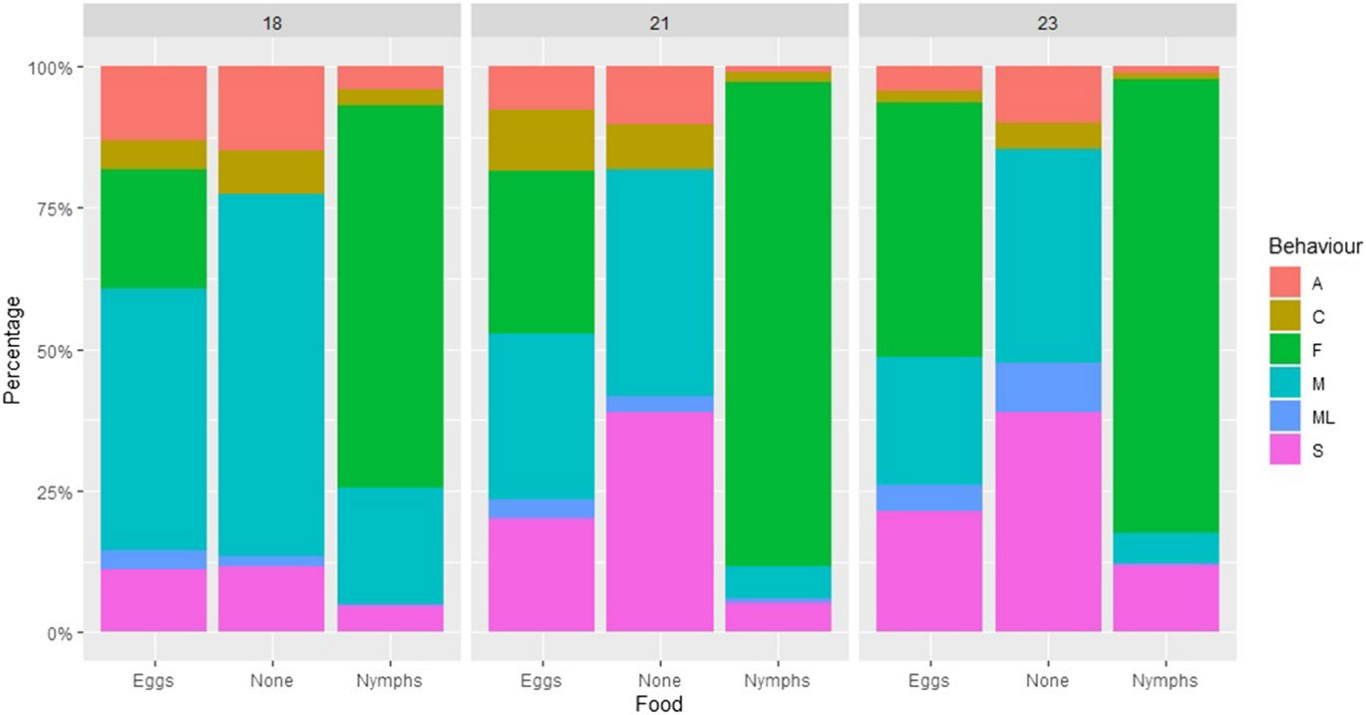
The percentage of time spent demonstrating six different behavior types (F- feeding, M- moving, ML- moving leaf, C- cleaning, S- stationary and A- antennating), depending on temperature treatment (18 °C, 21 and 23 °C) and food type (eggs, no-food and nymphs) for *Anthocoris nemoralis* for *Cacopsylla pyri* prey

On average 3.90 ± 4.70 eggs were eaten by *A. nemoralis* in the 18 °C treatment, compared to 5.70 ± 5.80 eggs and 7.65 ± 5.49 for the 21 and 23 °C treatments. However, the number of eggs eaten did not significantly differ depending on temperature (Kruskal Wallis: χ^2^ = 5.19, df = 2, *p* = 0.075). Sex also did not significantly impact the number of eggs eaten (Kruskal Wallis: χ^2^ = 0.498, df = 2, *p* = 0.481). The average amount of time spent feeding in the egg treatment was 252.5 ± 361.6 s in the 18 °C treatment, compared to 342.9 ± 402.2 s and 543.0 ± 468.1s for the 21 and 23 °C treatments (Fig. 4), ranging from 0 to 1200 s spent feeding. However, there was no significant difference in the amount of time spent feeding depending on temperature in the egg (Kruskal Wallis: χ^2^ = 4.90, df = 2, *p* = 0.086) or nymph (Kruskal Wallis: χ^2^ = 1.46, df = 2, *p* = 0.481) treatments. The amount of time spent feeding by *A. nemoralis* significantly differed depending on the food treatment (Kruskal Wallis: χ^2^ = 39.14, df = 1, *p* > 0.001), with an average of 379.5 ± 424.0s spent feeding in the egg treatment and 934.1 ± 438.6s spent feeding in the nymph treatment. There was no significant difference in time spent feeding depending on sex (Kruskal Wallis: χ^2^ = 0.003, df = 1, *p* = 0.955).

*Anthocoris nemoralis* spent significantly more time moving in the 18 °C treatment, compared to the 21 and 23 °C treatments, for no-food (Kruskal Wallis: χ^2^ = 9.57, df = 2, *p* = 0.008) and eggs (Kruskal Wallis: χ^2^ = 9.62, df = 2, *p* = 0.008) but not nymphs (Fig. 4). Anthocorids spent significantly more time cleaning at 18 and 21 °C compared to 23 °C the egg treatment (Kruskal Wallis: χ^2^ = 7.38, df = 2, *p* = 0.025). There was also a significant difference in the time spent antennating depending on temperature for the egg treatment (Kruskal Wallis: χ^2^ = 10.95, df = 2, *p* = 0.004), with more time spent antennating at 18 °C compared to 23 °C. For the no-food treatment, significantly more time was spent stationary (Kruskal Wallis: χ^2^ = 11.39, df = 2, *p* = 0.003) at 21 and 23 °C compared to 18 °C. However, there was no significant difference in the time spent on moving the leaf depending on temperature (Kruskal Wallis: χ^2^ = 5.06, df = 2, p 0.080), or time spent on any of the behaviors depending on sex.

## Discussion

### Functional Response of *A. nemoralis* to *C. pyri* Prey

For this study both male and female *A. nemoralis* exhibited Type II functional responses for all three temperature treatments tested, when feeding on hardshell (4th and 5th instar) *C. pyri* nymphs (Fig. 1). Thus, confirming the first hypothesis. A Type II functional response demonstrates that at lower prey densities the consumption rate of prey increases as prey density increases, but eventually levels off and remains constant at high densities (Real 1977; Juliano 2020). This corresponds to multiple other studies, where a Type II functional response was reported for adult anthocorid species of feeding on psyllid nymphs; including *A. minki* predating on *Psyllopsis repens* (Hassanzadeh-Avval et al. 2019), A. *nemoralis* on *C. pyricola* (Emami et al. 2014a), *A. minki pistaciae* on *Agonoscena pistaciae* (Kheradmand et al. 2017) and *Orius vicinus* on *Bactericera cockerelli* (Tran et al. 2012). A Type II functional response may be more optimal than a Type III response (where consumption rates are lower than Type II responses at low prey densities) for anthocorids, as biological control agents are able to detect and attack prey more effectively at low densities (Lopes et al. 2009). Although, Type II functional responses can be destabilizing in comparison to Type III; high consumption rates of prey at low prey densities can lead to prey population destabilization (Dick et al. 2014; Cuthbert et al. 2021). However, Type III responses allow a negative density-dependent response of the prey survival with prey population density, stabilizing prey populations and making them less likely to fluctuate (Cuthbert et al. 2021).

### Functional Response and Temperature

All three temperature treatments exhibited a Type II functional response. Although males within the 23 °C treatment had a shorter handling time than those at 18 °C, female handling time was not significantly impacted by temperatures tested in this study. Handling times may have decreased with temperature, due to reduced time required for digestion as a result of an increased metabolic rate (Sentis et al. 2013; Robertson and Hammill 2021). Furthermore, male *A. nemoralis* have a smaller body size than females (Coblentz et al. 2022), thus may be more sensitive to changes in temperature, resulting in a higher increase in metabolic rate (Frances and McCauley 2018). Other studies found decreases in handling time with temperature (Knutsen and Salvanes 1999; Sentis et al. 2013; Robertson and Hammill 2021), for example the spined soldier bug (*Podisus nigrispinus*) handling time for the prey species *Spodoptera exigua* decreased at higher temperatures (Clercq 2001).

However, there was not a significant difference in *A. nemoralis* attack rate depending on temperature for males or females within this study. Attack rates have been indicated to increase with rising temperature in other studies, demonstrating a hump-shaped response, with highest attack rates at intermediate temperatures (Uiterwaal and DeLong 2020; Robertson and Hammill 2021). The non-significant difference in temperature dependent attack rates, may be due to the small intervals between testing at different temperatures, therefore larger intervals between treatments may be required to detect differences. Many other functional response experiments use temperatures with higher intervals between them; for example, Hassanzadeh-Avval et al. (2019) used 15, 24 and 30 °C with the anthocorid *A. minki* and significant differences in attack rate were only seen between the 15 and 30 °C treatments. Whilst 20, 25 and 30 °C were temperature treatments used for functional responses of *Orius laevigatus* (Hassanpour et al. 2020), with a significantly higher attack rate of female anthocorids at 30 °C compared to 20 °C. Therefore, UK mean temperatures predicted for 2080, are unlikely to impact the feeding rates of anthocorids compared to current temperatures. However, *C. pyri* population growth and feeding rates have not been observed under these temperatures. Therefore, if pests respond differently compared to anthocorids, summer pear sucker populations could be difficult to control, highlighting an area of future research. In addition, maximum UK temperatures and future predicted temperatures for other pear growing regions have not been studied, thus future climate could still impact predator-pest dynamics at larger temperature intervals.

#### Functional Response and Sex of Anthocorid

This study found that female anthocorids had a significantly higher attack rate and shorter handling time than males, supporting the fourth hypothesis, that sex of anthocorid influences functional response. This is similar to the findings of Emami et al. (2014a), where *A. nemoralis* females had higher attack rates, maximal consumption rates and shorter handling times for *C. pyricola* nymphs than males. Hassanzadeh-Avval et al. (2019) also found the maximal consumption rate for ash psyllid, *P. repens*, L4 nymphs (a similar sized prey type to *C. pyri*) was higher for females than males, for the anthocorid *A. minki*. Therefore, female *A. nemoralis* may be a more effective biological control agent of *C. pyri* nymphs than males, although sex ratios of *A. nemoralis* are both 1:1 in summer (in pear orchards) and overwintering populations (McMullen and Jong 1967), so it is unlikely that this will have implications for pest control as numbers of females and males found in orchards are approximately equal.

The differences in attack rate and handling time depending on sex may be due to differences in body size as male *A. nemoralis* are smaller than females, with a lower body weight (Campbell 1977). Body size has a significant effect on the feeding rate of a predator (Aljetlawi et al. 2004; DeLong 2021; Robertson and Hammill 2021; Coblentz et al. 2022); handling time may decrease as predator body size increases as it may be easier to handle and subdue large prey items (Hammill et al. 2015; Robertson and Hammill 2021). Conversely, attack rate may increase with body size, as larger predators may cover more distance, increasing the chance of encountering prey (DeLong 2021), although difference in distance travelled depending on sex was not apparent in the behavioral assays. There is also the possibly that females have higher nutritional requirements than males (Coblentz et al. 2022), in order to produce eggs, therefore need to consume more prey, as females were likely mated within this study. This emphasizes the need for further research, exploring whether differences in prey consumption between males and females is sex dependent or size dependent.

#### Behavioral Assays

##### Changes in *A. nemoralis* Activity and Behavior in Response to Temperature

Results from the behavioural assays found that anthocorids spent less time moving at higher temperatures (Fig. 4). When no prey or only eggs were available, anthocorids spent more time moving at 18 °C, compared to 21 and 23 °C. There was also less time spent antennating and cleaning at higher temperatures in the no-food or egg treatments. For no-food treatment, significantly more time was spent stationary at high temperatures compared to low. This suggests that *A. nemoralis* may spend more time active at current UK temperatures (18 °C) compared to future temperature regimes, as 23 °C resulted in the lowest activity levels.

However, despite less time spent active at higher temperatures, there was no difference in velocity or distance travelled overall, suggesting that the speed of the anthocorid when it was moving may be faster at higher temperatures. Hence, anthocorids can travel similar distances, suggesting they will be efficient predators at high temperatures tested here; as a high walking velocity is important for a biological control agent (Milton 2004), allowing a predator to seek out prey items effectively, especially if prey is sparse. Our study also revealed a reduction in cleaning behavior at the highest temperature treatment in the egg treatment. A reduction in cleaning behavior may increase the risk of disease; grooming is an important sanitary behavior, involved in reducing the risk of pathogenic infection, including parasites and fungal pathogens (De Roode and Lefèvre 2012; Zhukovskaya et al. 2013). However, more research is required to see if reduced grooming in anthocorids increases disease risk, as increased disease risk could reduce the efficiency of a biological control agent.

#### Anthocorid Feeding Behavior and Prey Preference

There was no significant difference in the time spent feeding at each of the three temperature treatments (for eggs and nymph treatments) or number of eggs eaten (Fig. 4). This suggests that temperature did not significantly affect the rate of feeding for these temperature intervals. Time spent feeding was higher for nymphs compared to eggs, furthermore there was a far higher number of replicates where 0 eggs were eaten, compared to replicates where 0 nymphs were eaten, suggesting a preference for *C. pyri* (L4 – L5 nymphs) compared to eggs. Conversely, the study by Sigsgaard (2010), found no significant preference between *C. pyri* eggs or nymphs depending on biomass for *A. nemoralis*; however their study used L1-L3 nymphs rather than L4-L5, perhaps larger darker nymphs, are easier to find than eggs for *A. nemoralis*. In addition, there could be a difference in amount or type of volatiles given off by different stages of *C. pyri* nymphs and eggs, making nymphs easier to detect via olfactory cues. Although, there is evidence for volatile emission in pear sucker adults (Ganassi et al. 2018), no studies have occurred for other stages of *C. pyri*, emphasizing the need for further research. Despite the differences in attack rate and handling time depending on sex of anthocorid, there was no difference in time spent feeding, number of eggs eaten or any other variables monitored in our behavioral assays, possibly due to the short, 20-minute, duration of our assays with few prey items, whereas the functional response experiments occurred over 24 h at larger prey densities. Therefore, perhaps assays over a longer time period are required to observe sex specific differences.

## Conclusions

The anthocorid, *A. nemoralis*, is likely to be an effective predator of *C. pyri* nymphs under future predicted temperatures, within the UK. Attack rates and overall prey consumption by anthocorids did not significantly differ depending on temperature, although males did show significantly shorter handling times at 23 °C compared to 18 °C. However, it is important to note that only UK average summer temperatures (current and predicted) were monitored under laboratory conditions, therefore looking at functional responses for a larger temperature range may be necessary for other pear growing regions. Despite the likelihood that anthocorid feeding rates will be similar for current and future UK summer temperatures, there is still the possibility that pear sucker feeding and growth rates could change. The body size of pear sucker nymphs and adults are smaller than anthocorids, making them more sensitive to changes in temperature, which could lead to increased feeding and growth rates. Therefore, if there is a mismatch between pear sucker and anthocorid population growth or feeding rates, it may be more difficult to control summer pest populations. This highlights the importance of monitoring multiple trophic levels within an agricultural ecosystem (primary producer, pest and natural enemy), to observe interactions and potential mismatches between them. This study suggests that future research should focus on these trophic interactions using mesocosm studies, field or glasshouse experiments.

## Supplementary Information

Below is the link to the electronic supplementary material.

ESM 1 (DOCX 43.1 KB)

## Data Availability

Dataset accessible from the University of Reading Research Data Archive.

## References

[CR1] Aartsma Y, Cusumano A, De Bobadilla MF, Rusman Q, Vosteen I, Poelman EH (2019). Understanding insect foraging in complex habitats by comparing trophic levels: insights from specialist host-parasitoid-hyperparasitoid systems. Curr Opin Insect Sci.

[CR2] Agroscience R (2022) Psyllid key. RLP AgroScience GmbH. https://agroscience.de/index.php/de/kernthemen/anwendungen-der-digitalisierung/psyllidkey. Accessed 17 Nov 2022

[CR3] AHDB (2012) Final Report-Exploiting semiochemicals, conservation biocontrol and selective physical controls in integrated management of pear sucker. https://projectbluearchive.blob.core.windows.net/media/Default/Research%20Papers/Horticulture/TF%20181%20final%202012%20psg.pdf. Accessed 11 Sept 2022

[CR4] Aljetlawi AA, Sparrevik E, Leonardsson K (2004). Prey–predator size-dependent functional response: derivation and rescaling to the real world. J Anim Ecol.

[CR5] Barford E (2013). Crop pests advancing with global warming. Nature.

[CR6] Beninato S, Morella S (2000). Control of *Cacopsylla pyri* with massive releases of *Anthocoris nemoralis* in pear orchards. Atti Gior Fitopatolog.

[CR7] Boege K, Agrawal AA, Thaler JS (2019). Ontogenetic strategies in insect herbivores and their impact on tri-trophic interactions. Curr Opin Insect Sci.

[CR8] Boggs CL (2016). The fingerprints of global climate change on insect populations. Curr Opin Insect Sci.

[CR9] Bolker BM (2008). Ecological models and data in R. Ecological Models and Data in R.

[CR10] BPDB (2022) *Anthocoris nemoralis*. Bio-Pesticides DataBase. http://sitem.herts.ac.uk/aeru/bpdb/Reports/2087.html. Accessed 11 Sept 2022

[CR11] Bu KY (2007). Female copulatory tubes and the subdivision of the genus *Anthocoris* (Heteroptera: Anthocoridae: Anthocorini). Eur J Entomol.

[CR12] Bulgarini G, Badra Z, Leonardi S, Maistrello L (2021). Predatory ability of generalist predators on eggs, young nymphs and adults of the invasive *Halyomorpha halys* in southern Europe. Dordr.

[CR13] Campbell C (1977). Alaboratory evaluation of *Anthocoris nemorum* and *A. nemoralis* [Hem.: Anthocoridae] as predators of *Phorodon humuli* [Hom.: Aphididae]. Entomophaga.

[CR14] Campolo O, Malacrinò A, Laudani F, Maione V, Zappalà L, Palmeri V (2014). Population dynamics and temperature-dependent development of *Chrysomphalus aonidum* (L.) to aid sustainable pest management decisions. Neotrop Entomol.

[CR15] Carraro L, Loi N, Ermacora P (2001). The ‘life cycle’ of pear decline phytoplasma in the vector *Cacopsylla pyri*. J Plant Pathol.

[CR16] Chen YH, Gols R, Benrey B (2015). Crop domestication and its impact on naturally selected trophic interactions. Annu Rev Entomol.

[CR17] Chireceanu C (1998). Biometrical investigations in Pear Psyllids *Cacopsylla Pyri* and *C. pyrisuga* (Homoptera: Psyllidae) populations in Baneasa Bucharest area. Entomol rom.

[CR18] Clercq D (2001). Functional response of the predators *Podisus maculiventris* (say) and *Podisus nigrispinus* (Dallas)(Het., Pentatomidae) to the beet armyworm, *Spodoptera exigua* (Hübner)(Lep., Noctuidae): effect of temperature. J Appl Entomol.

[CR19] Coblentz KE, Squires A, Uiterwaal S, Delong JP (2022). Quantifying predator functional responses under field conditions reveals interactive effects of temperature and interference with sex and stage. J Anim Ecol.

[CR20] Cuthbert RN, Dalu T, Wasserman RJ, Sentis A, Weyl OL, Froneman PW, Dick JT (2021). Prey and predator density-dependent interactions under different water volumes. Ecol Evol.

[CR21] Daniel C, Pfammatter W, Kehrli P, Wyss E (2005). Processed kaolin as an alternative insecticide against the european pear sucker, *Cacopsylla pyri* (L). J Appl Entomol.

[CR22] DeLong JP (2021) Predator ecology: evolutionary ecology of the functional response. Oxford Academic, Oxford, pp 1–167. 10.1093/oso/9780192895509.001.0001

[CR23] Dick JT, Alexander ME, Jeschke JM, Ricciardi A, MacIsaac HJ, Robinson TB, Hatcher MJ (2014). Advancing impact prediction and hypothesis testing in invasion ecology using a comparative functional response approach. Biol Invasions.

[CR24] Ding-Xu L, Juan T, Zuo-Rui S (2007). Functional response of the predator Scolothrips takahashii to hawthorn spider mite, *Tetranychus viennensis*: effect of age and temperature. Biocontrol.

[CR25] Duffy C (2014) Developing a temperature-dependent simulation model for Sitobion avenae: Impacts of climate change for spring barley in Ireland. Dissertation, National University of Ireland, Maynooth (Ireland)

[CR26] Duffy GA, Coetzee BW, Janion-Scheepers C, Chown SL (2015). Microclimate-based macrophysiology: implications for insects in a warming world. Curr Opin Insect Sci.

[CR27] Emami MS, Shishehbor P, Karimzadeh Esfahani J (2014). Functional response of *Anthocoris nemoralis* (Hemiptera: Anthocoridae) to the pear psylla, *Cacopsylla pyricola* (Hemiptera: Psyllidae): effect of pear varieties. J Crop Prot.

[CR28] Emami MS, Shishehbor P, Karimzadeh J (2014). The influences of plant resistance on predation rate of *Anthocoris nemoralis* (Fabricius) on *Cacopsylla pyricola* (Förster). Arch Phytopathol Pflanzenschutz.

[CR29] Englund G, Öhlund G, Hein CL, Diehl S (2011). Temperature dependence of the functional response. Ecol Lett.

[CR30] Erler F (2004). Natural enemies of the pear psylla *Cacopsylla pyri* in treated vs untreated pear orchards in Antalya. Turk Phytoparasitica.

[CR31] Farhadi R, Allahyari H, Juliano SA (2010). Functional response of larval and adult stages of *Hippodamia variegata* (Coleoptera: Coccinellidae) to different densities of *Aphis fabae* (Hemiptera: Aphididae). Environ Entomol.

[CR32] Fields G, Beirne B (1973) Ecology of anthocorid (Hemiptera: Anthocoridae) predators of the pear psylla (Homoptera: Psyllidae) in the Okanagan Valley, British Columbia. J Entomol Soc BC 70:18–19

[CR33] Frances DN, McCauley SJ (2018). Warming drives higher rates of prey consumption and increases rates of intraguild predation. Oecologia.

[CR34] Gajski D, Pekár S (2021). Assessment of the biocontrol potential of natural enemies against psyllid populations in a pear tree orchard during spring. Pest Manag Sci.

[CR35] Ganassi S, Germinara GS, Pati S, Civolani S, Cassanelli S, Sabatini MA, De Cristofaro A (2018). Evidence of a female-produced sex pheromone in the european pear psylla *Cacopsylla pyri*. Bull Insectology.

[CR36] Hammill E, Atwood TB, Corvalan P, Srivastava DS (2015). Behavioural responses to predation may explain shifts in community structure. Freshw Biol.

[CR37] Hassanpour M, Yaghmaee A, Golizadeh A, Rafiee-Dastjerdi H, Mottaghinia L (2020). Functional response and consumption rate of *Orius laevigatus* (Hemiptera: Anthocoridae) feeding on the melon aphid *Aphis gossypii* (Hemiptera: Aphididae) at three different temperatures. J Crop Prot.

[CR38] Hassanzadeh-Avval M, Sadeghi-Namaghi H, Fekrat L (2019). Factors influencing functional response, handling time and searching efficiency of *Anthocoris minki* Dohrn (Hem.: Anthocoridae) as predator of psyllopsis repens Loginova. (Hem: Psyllidae) Phytoparasitica.

[CR39] Hassanzadeh-Avval M, Sadeghi-Namaghi H, Fekrat L (2020). Molecular and morphological identification of *Anthocoris* spp.(Hemiptera: Anthocoridae) predators of three economically important psyllid species in Razavi Khorasan province, northeastern Iran. Biologia.

[CR40] Holling CS (1965). The functional response of predators to prey density and its role in mimicry and population regulation. Mem Ent Soc Can.

[CR41] Jeschke JM, Kopp M, Tollrian R (2004). Consumer-food systems: why type I functional responses are exclusive to filter feeders. Biol Rev.

[CR42] Juliano SA (2020) Nonlinear curve fitting: predation and functional response curves. In Design and analysis of ecological experiments. 2nd edn, Chapman and Hall, London, pp 159–184

[CR43] Juliano S, Scheiner S, Gurevitch J (2001) Design and analysis of ecological experiments. Nonlinear curve fitting: predation and functional response curves, pp 178–196

[CR44] Kapatos E, Stratopoulou E (1999). Duration times of the immature stages of *Cacopsylla pyri* L.(Hom., Psyllidae), estimated under field conditions, and their relationship to ambient temperature. J Appl Entomol.

[CR45] Kheradmand K, Pourali Z, Jamshidnia A (2017). Influence of temperature on the functional response of the predatory bug, *Anthocoris minki pistaciae* (Hemiptera: Anthocoridae), a predator of *Agonoscena pistaciae* (Hemiptera: Psyllidae). Zool Ecol.

[CR46] Kindlmann P, Dixon A, Dostálková I (2001). Role of ageing and temperature in shaping reaction norms and fecundity functions in insects. J Evol Biol.

[CR47] Knutsen I, Salvanes AGV (1999). Temperature-dependent digestion handling time in juvenile cod and possible consequences for prey choice. Mar Ecol Prog Ser.

[CR48] Kucerová J, Talacko L, Lauterer P, Navratil M, Fialová R (2007). Molecular tests to determine *Candidatus Phytoplasma pyri* presence in psyllid vectors from a pear tree orchard in the Czech Republic–a preliminary report. Bull Insectol.

[CR49] Ladányi M, Horváth L (2010). A review of the potential climate change impact on insect populations- general and agricultural aspects. Appl Ecol Environ Res.

[CR50] Liu J, Wang C, Desneux N, Lu Y (2021). Impact of temperature on survival rate, fecundity, and feeding behavior of two aphids, *Aphis gossypii* and *Acyrthosiphon gossypii*, when reared on cotton. Insects.

[CR51] Lopes C, Spataro T, Lapchin L, Arditi R (2009). Optimal release strategies for the biological control of aphids in melon greenhouses. Biol Control.

[CR52] May ML (1979). Insect thermoregulation. Annu Rev Entomol.

[CR53] McMullen R, Jong C (1967). New records and discussions of predators of the pear psylla, *psylla pyricola* Forster, in British Columbia. J Ent Soc Br Columb.

[CR54] McMullen R, Jong C (1972). Influence of temperature and host vigor on fecundity of pear psylla (Homoptera: Psyllidae) 1. Can Entomol.

[CR55] Mellanby K (1939). Low temperature and insect activity. Proc Royal Soc B.

[CR56] Milonas PG, Kontodimas DC, Martinou AF (2011). A predator’s functional response: influence of prey species and size. Biol Control.

[CR57] Milton B (2004) The influence of foliar pubescens on searching activity of the whitefly parasitoid *Encarsia formosa* Gahan (Hymenoptera: Aphelinidae) on different poinsettia (*Euphorbia pulcherrima* Willd. ex Koltz.) cultivars and temperatures. And The influence of temperature in parasitization of *Bemisia tabaci* Gennadius. Dissertation, The Agricultural University of Norway

[CR58] Montanari S, Guérif P, Ravon E, Denancé C, Muranty H, Velasco R, Perchepied L (2015). Genetic mapping of *Cacopsylla pyri* resistance in an interspecific pear (*Pyrus* spp.) population. Tree Genet Genomes.

[CR59] Nagy C, Cross J, Luton M, Ashdown C (2008). Mixed deciduous hedgerows as sources of anthocorids and other predators of pear psyllids in the UK. IOBC Conf Integrated Fruit Prod Avignon.

[CR60] Noldus LP, Spink AJ, Tegelenbosch RA (2001). EthoVision: a versatile video tracking system for automation of behavioral experiments. Behav Res Methods Instrum Comput.

[CR61] Noldus LP, Spink AJ, Tegelenbosch RA (2002). Computerised video tracking, movement analysis and behaviour recognition in insects. Comput Electron Agric.

[CR62] Oz V, Erler F (2021). Evaluation of oviposition deterrent activity of four oily substances against winterform females of pear psylla, Cacopsylla pyri. Bull Insectol.

[CR63] Pritchard DW, Paterson R, Bovy HC, Barrios-O’Neill D (2017). Frair: an R package for fitting and comparing consumer functional responses. Methods Ecol Evol.

[CR64] Ratte HT (1984) Temperature and insect development. Environmental physiology and biochemistry of insects. Springer, Berlin, Heidelberg, pp 33–66. 10.1007/978-3-642-70020-0_2

[CR65] Real LA (1977). The kinetics of functional response. Am Nat.

[CR66] Robertson ML, Hammill E (2021). Temperature and prey morphology influence attack rate and handling time in a predator–prey interaction. Hydrobiologia.

[CR67] Rogers D (1972). Random search and insect population models. J Anim Ecol.

[CR68] De Roode JC, Lefèvre T (2012). Behavioral immunity in insects. Insects.

[CR69] Sable M, Rana D (2016). Impact of global warming on insect behavior-A review. Agric Rev.

[CR70] Salvianti F, Bettini PP, Giordani E, Sacchetti P, Bellini E, Buiatti M (2008). Identification by suppression subtractive hybridization of genes expressed in pear (*Pyrus* spp.) upon infestation with *Cacopsylla pyri* (Homoptera: Psyllidae). J Plant Physiol.

[CR71] Saour G, Ismail H, Hashem A (2010). Impact of kaolin particle film, spirodiclofen acaricide, harpin protein, and an organic biostimulant on pear psylla *Cacopsylla pyri* (Hemiptera: Psyllidae). Int J Pest Manag.

[CR72] Schaub L, Graf B, Butturini A (2005). Phenological model of pear psylla *Cacopsylla pyri*. Entomol Exp Appl.

[CR73] Schmitz OJ, Barton BT (2014). Climate change effects on behavioral and physiological ecology of predator-prey interactions: implications for conservation biological control. Biol Control.

[CR74] Scutareanu P, Lingeman R, Drukker B, Sabelis MW (1999) Cross‐correlation analysis of fluctuations in local populations of pear psyllids and anthocorid bugs. Ecol Entomol 24(3):354–363. 10.1046/j.1365-2311.1999.00199.x

[CR75] Sek Kocourek F, Stará J (2006). Management and control of insecticide-resistant pear psylla (*Cacopsylla pyri*). J Fruit Ornam Plant Res.

[CR76] Sentis A, Hemptinne JL, Brodeur J (2013). Parsing handling time into its components: implications for responses to a temperature gradient. Ecology.

[CR77] Shaltiel L, Coll M (2004). Reduction of pear psylla damage by the predatory bug *Anthocoris nemoralis* (Heteroptera: Anthocoridae): the importance of orchard colonization time and neighboring vegetation. Biocontrol Sci Technol.

[CR78] Shaw B, Nagy C, Fountain MT (2021). Organic control strategies for use in IPM of invertebrate pests in apple and pear orchards. Insects.

[CR79] Sigsgaard L (2010). Habitat and prey preferences of the two predatory bugs Anthocoris nemorum (L.) and A. nemoralis (Fabricius) (Anthocoridae: Hemiptera-Heteroptera). Biol Control.

[CR80] Solomon M, Cross J, Fitzgerald J, Campbell C, Jolly R, Olszak R, Vogt H (2000). Biocontrol of pests of apples and pears in northern and central Europe-3. Predators. Biocontrol Sci Technol.

[CR81] South J, Welsh D, Anton A, Sigwart J, Dick J (2018). Increasing temperature decreases the predatory effect of the intertidal shanny *Lipophrys pholis* on an amphipod prey. J Fish Biol.

[CR82] Süle S, Jenser G, Szita E, Bertaccini A, Maini S (2007). Management of pear decline caused by ‘*Candidatus Phytoplasma* pyri’in Hungary. Bull Insectol.

[CR83] Tobin PC, Nagarkatti S, Loeb G, Saunders MC (2008). Historical and projected interactions between climate change and insect voltinism in a multivoltine species. Glob Chang Biol.

[CR84] Tran L, Worner S, Vereijssen J, Teulon D (2012) Population dynamics of tomato and potato psyllid (*Bactericera cockerelli*) in New Zealand. Zebra Chip Reporting Session. https://www.researchgate.net/profile/Tariq-Mustafa-2/publication/276934059_Effects_of_Host_Plant_on_Development_and_Body_Size_of_Three_Haplotypes_of_Bactericera_cockerelli_Hemiptera_Triozidae/links/562dcb5e08ae04c2aeb4a9e2/Effects-of-Host-Plant-on-Development-and-Body-Size-of-Three-Haplotypes-of-Bactericera-cockerelli-Hemiptera-Triozidae.pdf#page=25. Accessed 19 Nov 2022

[CR85] Uiterwaal SF, DeLong JP (2018). Multiple factors, including arena size, shape the functional responses of ladybird beetles. J Appl Ecol.

[CR86] Uiterwaal SF, DeLong JP (2020). Functional responses are maximized at intermediate temperatures. Ecology.

[CR87] UKCP (2021) UK Climate Projections User Interface Data: Anomalies for probabilistic projections (25km) over UK, 1961–2100. https://ukclimateprojections-ui.metoffice.gov.uk/products/form/LS1_Sample_01 Accessed 11 Sept 2022

[CR88] Wojda I (2017). Temperature stress and insect immunity. J Therm Biol.

[CR89] Yanik E, Ugur A (2004). Avcı böcek *Anthocoris nemoralis* (F.)(Heteroptera: Anthocoridae)’in laboratuvar ve doğa şartlarında *Cacopsylla pyri* (L.)(Homoptera: Psylidae) ve *Ephestia kuehniella* Zell.(Lepidoptera: Pyralidae) yumurta tüketimi. Bitki Koruma Bülteni.

[CR90] Yuan JS, Himanen SJ, Holopainen JK, Chen F, Stewart CN (2009). Smelling global climate change: mitigation of function for plant volatile organic compounds. Trends Ecol Evol.

[CR91] Zhukovskaya M, Yanagawa A, Forschler BT (2013). Grooming behavior as a mechanism of insect disease defense. Insects.

[CR92] Zidon R, Tsueda H, Morin E, Morin S (2016). Projecting pest population dynamics under global warming: the combined effect of inter-and intra‐annual variations. Ecol Appl.

