# Supplementary material for: Functional and Behavioral Responses of the Natural Enemy Anthocoris nemoralis to Cacopsylla pyri, at Different Temperatures
Source: J Insect Behav. 2023 Jul 7;36(3):222–38. doi: 10.1007/s10905-023-09836-5 (PMC10403413; doi:10.1007/s10905-023-09836-5)
Supplement: Supplementary file 1 — (DOCX 43.1 KB) [file 10905_2023_9836_MOESM1_ESM.docx]

Supplementary material:

**Table S1** Kruskal-Wallis tests of the significance in difference in behaviour (feeding, moving, moving leaf, cleaning, stationary and antennating), time spent in zone, number of eggs eaten, velocity and distance travelled for *A. nemoralis* depending on batch number.

| Observation/behaviour | df | χ^2^ | P value |
| --- | --- | --- | --- |
| Feeding | 4 | 5.96 | 0.201 |
| Cleaning | 4 | 10.39 | 0.0342 |
| Antennating | 4 | 8.48 | 0.0755 |
| Moving | 4 | 9.39 | 0.0522 |
| Moving leaf | 4 | 6.48 | 0.166 |
| Velocity | 4 | 4.33 | 0.362 |
| Distance travelled | 4 | 4.49 | 0.343 |
| Time spent in Centre zone | 4 | 5.62 | 0.229 |

**Table S2** The July-August mean temperature predicted for the RCP 4.5 and RCP 8.5 emissions scenarios in 2080 (±SD), as well as the current July-August mean temperature (1990-2020). Also shown are the scenarios rounded to the nearest degree and the temperature/ relative humidity recorded in each of the controlled temperature cabinets during the study.

| Scenario | Temp Scenario (˚C) | Temp rounded (˚C) | Temp recorded (˚C) | Humidity recorded (RH) |
| --- | --- | --- | --- | --- |
| Current | 17.78 ± 2.53 | 18 | 18.54 ± 0.72 | 22.41 ± 6.95 |
| RCP 4.5 | 20.57 ± 2.39 | 21 | 21.40 ± 0.59 | 25.92 ± 7.74 |
| RCP 8.5 | 22.55 ± 3.05 | 23 | 22.92 ± 0.34 | 24.72 ± 5.29 |

**Table S3** 95% confidence intervals (CIs), provided nonparametric bootstrapping. CIs provided for three different temperature treatments (18°C, 21 °C and 23 °C), for male and female *A. nemoralis*.

| Temp (°C) | Sex | Coeff | CI (lower) | CI (upper) |
| --- | --- | --- | --- | --- |
| 18 | M | $a$ | 0.033 | 0.152 |
|  |  | $h$ | 2.17 | 4.01 |
| 21 | M | $a$ | 0.041 | 0.120 |
|  |  | $h$ | 2.40 | 3.64 |
| 23 | M | $a$ | 0.034 | 0.077 |
|  |  | $h$ | 1.86 | 2.82 |
| 18 | F | $a$ | 0.078 | 0.157 |
|  |  | $h$ | 1.53 | 2.20 |
| 21 | F | $a$ | 0.113 | 0.216 |
|  |  | $h$ | 1.60 | 2.03 |
| 23 | F | $a$ | 0.093 | 0.197 |
|  |  | $h$ | 1.39 | 1.92 |
